# Enhancing Fetal Electrocardiogram Signal Extraction Accuracy through a CycleGAN Utilizing Combined CNN–BiLSTM Architecture

**DOI:** 10.3390/s24092948

**Published:** 2024-05-06

**Authors:** Yuyao Yang, Lin Chen, Shuicai Wu

**Affiliations:** Department of Biomedical Engineering, College of Chemistry and Life Science, Beijing University of Technology, Beijing 100124, China; yoga3y@emails.bjut.edu.cn (Y.Y.); chenlin_2023@emails.bjut.edu.cn (L.C.)

**Keywords:** fetal electrocardiogram signal extraction, CycleGAN, convolutional neural networks, bidirectional long short-term memory, PatchGAN

## Abstract

The fetal electrocardiogram (FECG) records changes in the graph of fetal cardiac action potential during conduction, reflecting the developmental status of the fetus in utero and its physiological cardiac activity. Morphological alterations in the FECG can indicate intrauterine hypoxia, fetal distress, and neonatal asphyxia early on, enhancing maternal and fetal safety through prompt clinical intervention, thereby reducing neonatal morbidity and mortality. To reconstruct FECG signals with clear morphological information, this paper proposes a novel deep learning model, CBLS-CycleGAN. The model’s generator combines spatial features extracted by the CNN with temporal features extracted by the BiLSTM network, thus ensuring that the reconstructed signals possess combined features with spatial and temporal dependencies. The model’s discriminator utilizes PatchGAN, employing small segments of the signal as discriminative inputs to concentrate the training process on capturing signal details. Evaluating the model using two real FECG signal databases, namely “Abdominal and Direct Fetal ECG Database” and “Fetal Electrocardiograms, Direct and Abdominal with Reference Heartbeat Annotations”, resulted in a mean MSE and MAE of 0.019 and 0.006, respectively. It detects the FQRS compound wave with a sensitivity, positive predictive value, and *F*_1_ of 99.51%, 99.57%, and 99.54%, respectively. This paper’s model effectively preserves the morphological information of FECG signals, capturing not only the FQRS compound wave but also the fetal P-wave, T-wave, P-R interval, and ST segment information, providing clinicians with crucial diagnostic insights and a scientific foundation for developing rational treatment protocols.

## 1. Introduction

Congenital heart disease (CHD) is the leading cause of stillbirths worldwide, and it is the most common major congenital malformation [1,2]. The emergence of this defect is typically noted in the early stages of fetal heart formation. Prenatal monitoring and timely diagnosis are imperative for effectively addressing these conditions and minimizing potential complications, thereby decreasing fetal morbidity and mortality [3]. Cardiotocography (CTG) is currently the most widely used electronic fetal monitoring (EFM) device in clinical practice [4]. This method involves the transmission of ultrasonic waves through ultrasonic probes, followed by the reception of frequency-shift echo signals. Subsequently, the fetal cardiac cycle and uterine artery pulse index are computed, enabling the derivation of fetal heart rate and contraction curves. While this approach is robust and reliable, it poses challenges in capturing the variation information on instantaneous fetal heart rate. Additionally, the equipment’s size hinders its suitability for remote monitoring in a home setting. The fetal electrocardiogram (FECG) signal records the variations in fetal heart action potential during the conduction process. This enables the provision of beat-by-beat information on fetal heart rate as well as minute potential changes in the fetal heart activity cycle. Consequently, it equips doctors with essential information about the fetal health status, including indicators such as intrauterine hypoxia and fetal distress. This detailed data from the FECG signals enhance the ability to monitor and assess the well-being of the fetus during pregnancy [5,6]. In comparison with the traditional CTG method, the FECG signals stands out for its capacity to more comprehensively depict the overall scenario of fetal heart activity. This capability positions the FECG signals as the developmental trend in fetal monitoring for the future [7].

However, FECG signals are not extensively utilized in clinical practice presently, and this can be attributed to two main reasons. First, there is a shortage of experience in the clinical application of fetal electrocardiogram signals, coupled with a lack of standardized waveform recognition and databases. Second, the signal-to-noise ratio and amplitude of fetal electrocardiogram signals are relatively low, presenting challenges in preserving clear and comprehensive morphological information. There are two primary methods for acquiring FECG signals: invasive FECG (I-FECG) signal acquisition and non-invasive FECG (NI-FECG) signal acquisition [8]. The I-FECG signal acquisition method allows for the direct retrieval of high-quality FECG signals from the fetal scalp. However, this approach is limited to measurement during delivery, and its invasive nature introduces the risk of infection [9]. Therefore, in order to achieve long-term monitoring of fetal health status during the perinatal period, the NI-FECG signal acquisition method becomes essential. In this approach, FECG signals can be extracted by capturing maternal abdominal electrocardiograph (AECG) signals. The AECG signal is often contaminated by the maternal electrocardiograph (MECG), baseline drift, powerline interference, and pulse artifacts during acquisition [10,11]. The overlap of MECG signals and noise presents challenging issues in detecting the fetal QRS (FQRS) compound wave and preserving morphological information, such as P-waves, T-waves and ST segments [12].

In recent years, numerous advanced signal processing methods and noise filtering techniques have been employed in the extraction of FECG signals. These primarily encompass adaptive noise cancellation (ANC), singular-value decomposition (SVD), extended state Kalman filters (EKF), and blind source separation (BSS).

ANC is a filtering method grounded in linear filtering principles. It distinguishes itself from traditional signal processing methods by permitting spectral overlap between the target signal and the noise signal [13]. This algorithm automatically adjusts the filter parameters in real-time during the iteration process. It utilizes error signals based on the parameter results obtained from the previous moment, following some predefined criteria. This iterative adjustment aims to optimize the statistical characteristics of both the target signal and noise signal, ultimately achieving optimal filtering [14]. Various types of ANC algorithms exist, with the least mean square (LMS) and recursive least square (RLS) standing out as the two most commonly utilized algorithms in FECG signal extraction [15]. However, both of the mentioned algorithms fail to effectively strike a balance between convergence speed and steady-state error. Moreover, both ANC algorithms necessitate MECG signals as a reference. The practicality of this algorithm in practical clinical and remote monitoring is limited [16].

The EKF is an extension of the standard Kalman filter for nonlinear systems. It depends on the local linearization of the nonlinear model achieved by employing the Jacobian operator [17]. The EKF proves to be a robust method for the extraction of single-channel FECG signals [18]. Indeed, the performance of the EKF algorithm is contingent on local linearity. If the EKF encounters situations where the local linear assumption is violated, particularly in strongly nonlinear conditions, and the neglected high-order terms in the Taylor expansion result in significant errors, the EKF algorithm can lead to filtering divergence. This limitation renders the algorithm highly dependent on the positioning of the R-peak in the FECG signals during FECG signal extraction. When the maternal QRS compound wave overlaps with the fetal R-peak, issues related to waveform loss may arise in the extracted FECG signals.

SVD is a spatial filtering and decomposition technique that creates the required basis functions from the data itself and separates the statistics by maximizing the signal [19]. The algorithm is based on matrix transformation from one vector space to another, and when it is applied to FECG signal extraction, the SVD algorithm can effectively separate the components of the mixed signals, construct the vector matrix using the AECG signals, and then obtain the estimation of the ECG signals corresponding to each singular value via SVD. However, the SVD algorithm is only applicable in scenarios where the signal-to-noise ratio (SNR) of the FECG signals is high. Otherwise, it may introduce considerable noise into the separated FECG signals, leading to a significant decrease in the algorithm’s extraction performance.

Most BSS technologies are developed based on the principles of principal component analysis (PCA) and independent component analysis (ICA) [20,21]. Among them, the PCA algorithm focuses on reducing dimensionality in variable value measurement. In the process of simplifying statistical problems, PCA aims to retain the maximum amount of information and minimize information loss. This method can also be employed to identify linear combinations of discrete signals in statistics. It confirms data through bidirectional operations in a new coordinate system, ensuring no information loss throughout the entire process. However, when applying this algorithm to extract FECG signals, it may struggle to preserve the morphological information of FECG signals. The ICA algorithm is used to process the multichannel output data in order to estimate the optimal transmission matrix and obtain statistically significant mutually independent source components from it [22]. This algorithm has been successful in decomposing AECG signals into statistically independent MECG and FECG signals, even without a priori knowledge of the signals themselves. However, it is worth noting that the algorithm is sensitive to the initial weight vectors and is not guaranteed to achieve convergence in all cases.

The conventional techniques mentioned earlier for extracting FECG signals necessitate manual feature extraction, leading to incomplete noise removal in the extracted FECG signals. In recent years, the advent of advanced hardware has facilitated the widespread application of deep learning, yielding promising outcomes in FECG signal extraction. Compared with traditional algorithms reliant on manually designed feature extractors, deep learning models offer a significant advantage by autonomously learning and extracting intricate features from FECG signals. Achieved through the construction of multilayer neural network architectures, these models capture a wealth of detailed information inherent in the signals. Moreover, deep learning models exhibit high adaptability, facilitating self-optimization and adjustment to varying FECG signal characteristics [23]. The application of deep learning hinges on ample training data, rendering them robust against noise and interference. Notably, the features acquired during the training phase extend beyond applicability solely to the training dataset; they demonstrate robust generalization to unseen data. Thus, deep learning models proficiently process novel FECG signals without necessitating additional parameter adjustments or optimizations, even under conditions with a low signal-to-noise ratio. In this paper, we use an innovative deep learning model to extract FECG signals; the major contributions of the proposed work are depicted below:

- An unsupervised cycle generative adversarial network (CycleGAN) can effectively preserve the morphological information of FECG signals. The extracted FECG signals not only emphasize the FQRS compound wave but also capture the fetal P-wave and T-wave, PR intervals, and ST segment information.
- An innovative generator, utilizing both convolutional neural networks (CNN) and bidirectional long short-term memory (BiLSTM) during the feature extraction stage, effectively preserves the spatial and temporal characteristics of data, respectively.
- An innovative three-dimensional trajectory image is employed to visually represent FECG signal waveforms, utilizing cyclic consistency for subjective visual result evaluation.
- Two different real-world databases, “Abdominal and Direct FECG (A&D FECG)” and “NI-FECG PhysioNet2013”, demonstrated the effectiveness of the proposed BiLSTM–CNN CycleGAN for FECG signal extraction.

## 2. Related Works

Encoding–decoding networks have found extensive applications in the field of FECG signal extraction. In this approach, AECG signals undergo processing through an encoder, gradually reducing spatial dimensions while extracting relevant features. Subsequently, the FECG signal output is achieved by upsampling the features through a decoder.

Zhong et al. [24] developed a deep learning model for FECG signal extraction using a residual convolutional encoder–decoder network (RCED-Net). This model comprises five Conv–Deconv blocks, with shortcut connections employed between adjacent Conv–Deconv blocks. Consequently, details of the feature map can be directly passed from the top layers to the bottom layers, facilitating the flow of information and mitigating the vanishing gradient problem. Finally, the FECG signal is output through a fully connected layer. This method allows for the direct extraction of FECG signals from single-channel AECG signals without the need to eliminate MECG signals, thus avoiding the alignment registration problem associated with signal subtraction. However, it is worth noting that the complexity of the network model is relatively low, and its ability to extract complex AECG signals is considered insufficient.

The AECG-DecompNet framework, proposed by Rasti Meymandi-Arash et al. [25], comprises two residual symmetric hopping convolutional autoencoders (Res-Unet). AECG-DecompNet employs two distinct networks consecutively to decompose the AECG signal, one dedicated to MECG estimation and the other to interference and noise cancellation. Both networks employ an encoder–decoder architecture featuring internal and external hopping connections to augment signal reconstruction. AECG-DecompNet demonstrates the capability to extract both FECG and MECG signals from a single-channel AECG signal. Notably, it retains the ability to extract FECG signals with high quality even when the amplitude of FECG signals in the AECG signal is relatively low, enabling its application in the first trimester. However, training the sub-networks poses a challenge, given the necessity to train two separate network frameworks. In particular, there is a potential for error leakage from the first network to the second network.

Haiping Huang [26] proposes the temporal convolutional coding and decoding network (TCED-Net) to extract features of signals using 1D convolution. The network consists of a six-layer convolutional module and a corresponding inverse convolutional module, with residual and jump connections inside and outside, respectively, to enhance the end-to-end mapping of maternal ECG signals from the chest to the abdominal wall, and to apply the expansion convolution to perceive the signal features of longer historical moments. TCED-Net has superior nonlinear mapping ability, which is not limited to fetal heart rate estimation and QRS compound wave identification, but can effectively suppress the maternal ECG component and retain the morphological features of the FECG signal. Because it is difficult to collect the chest signals of pregnant women, this paper tries to use the maternal ECG template to replace the real chest ECG signal, which achieves better results and greatly reduces the discomfort of pregnant women and the difficulty of clinical examination.

Cycle generative adversarial network (CycleGAN) has received considerable interest in the domain of fetal electrocardiogram (FECG) signal extraction. In this approach, The generator is responsible for generating output data that align its features as closely as possible with the characteristics of the FECG signal based on the input AEGC signal. Meanwhile, the discriminator is responsible for determining whether the signal is a FECG signal generated by the generator or an authentic FECG signal. The model is implemented by alternately training the generator and the discriminator.

Mohebbian M R et al. [27] introduced an attention-based CycleGAN to map MECG and FECG signals. The novelty of this algorithm lies in the utilization of the attention mechanism as a filter mask to focus on the signal region of interest, the incorporation of a sinusoidal activation function, and log(cosh) loss, thereby preserving the morphological details of the FECG signal. Evaluation was conducted in a two-fold process: firstly, for the quality of FECG extracted from MECG, and secondly, for the detection of FQRS compound wave. The results were favorable in both evaluation methods. Despite the attention-based mechanism’s ability to obtain high-quality FECG signals, the model’s complexity is high, the running time is prolonged, and the computational cost is elevated, potentially posing challenges for embedded systems.

Wang X et al. [28] introduced a correlation-aware attention CycleGAN (CAA-CycleGAN) for the extraction of FECG signals. They developed three key modules: the auto-correlation attention encoder (ACAE) module, the cross-correlation attention residual (CCAR) module, and the dual-cross-correlation attention decoder (DCCAD) module. These modules were specifically designed for recovering FECG signals corrupted by noise, enhancing FECG components, and extracting FECG signals masked by the MECG signal. The algorithm’s innovation lies in incorporating a correlation attention network to enable the network to focus on the FQRS compound wave, thereby improving the detection capability of FQRS compound wave features. Nevertheless, the current implementation of the network appears to neglect other morphological information within FECG signals and has yet to address the computational overhead induced by the attention mechanism.

Basak P. et al. [29] utilized a 1D-CycleGAN to reconstruct FECG signals from MECG signals while preserving the morphology of the MECG signals. In the preprocessing stage, higher-order filters were chosen to enhance attenuation and narrow transition bands, surpassing the capabilities of traditional bandpass and bandstop filters for effective noise signal removal. Following signal inversion, the higher-order filter was reapplied to address any phase lag issues. For FECG signal extraction, weighted loss incorporating time, spectral, and power losses was employed, leading to a substantial enhancement in the quality of the generated FECG signals. This approach ensured the preservation of the complete signal morphology information, facilitating the accurate determination of fetal heart rate and heart rate variability indices. The performance of the 1D-CycleGAN in detecting FQRS compound waves exhibited a high accuracy, precision, recall, and *F*_1_ of 92.6%, 97.6%, 94.8%, and 96.4%, respectively. Nevertheless, there is potential for further improvement, particularly if the quality of the MECG signals is enhanced. Future enhancements could involve screening MECG signals to eliminate those of low quality or incorporating a module dedicated to improving MECG signal quality in the pre-processing stage.

Although the CycleGAN model achieves better results in extracting FECG signals, simply reducing the two-dimensional model to one-dimensional use will overlook the temporal features of the signal. In this paper, we propose a CycleGAN model that integrates CNN and BiLSTM (CBLS-CycleGAN) to incorporate temporal feature extraction alongside the original spatial feature extraction. This approach effectively preserves the morphological information of FECG signals and offers more clinically relevant insights.

## 3. Methodology

In this section, we will begin by introducing the databases used in this study. Following that, the methodology proposed in this study will be described in detail. Finally, the extraction process of the FECG signals will be briefly described.

### 3.1. Data Preparation

#### 3.1.1. Database Description

The data utilized in this study were sourced from three publicly available datasets.

The first database is the Abdominal and Direct Fetal Electrocardiogram Database (ADFECGDB), accessible at https://physionet.org/physiobank/database/ADFECGDB (accessed on 30 August 2022) [30]. The data were collected from five parturitions occurring at 38–41 weeks of gestation. Specifically, subject 1’s record is denoted as r01, subject 2’s record is denoted as r07, subject 3’s record is denoted as r10, subject 4’s record is denoted as r04, and subject 5’s record is denoted as r08. Each record includes four signals from the maternal abdomen and one signal directly from the fetal head. The sampling frequency is 1000 Hz, and the sampling time is 5 min.

The second database is the Fetal Electrocardiograms, Direct and Abdominal with Reference Heart Beats Annotations, accessible at https://springernature.figshare.com/articles/dataset/Fetal_electrocardiograms_direct_and_abdominal_with_reference_heart_beats_annotations/10311029?backTo=/collections/Fetal_electrocardiograms_direct_and_abdominal_with_reference_heart_beats_annotations/4740794 (accessed on 30 August 2023) [31]. The database comprises two datasets. The first dataset is the B1 Pregnancy dataset, denoted as the B1 pregnancy signal dataset, containing 10 records labeled “B1_Pregnancy_X”, where X represents the record number. Each record comprises 4 initially filtered AECG signals and 4 indirect FECG signals, acquired by suppressing the MECG signals through subtracting the first-order derivatives of the maternal P-QRS-T composite waveform and the QRS composite waveform. Each signal spans 20 min, with a sampling frequency of 500 Hz, stored in the binary file “B1_abSignals_X.ecg” in LabView format. Additionally, the file “B1_Maternal_R_X.txt” provides details regarding the maternal reference point, marking the position of the MQRS complex wave in the AECG signal. The file “B1_Fetal_R_X.txt” contains information on the fetal reference point, indicating the position of the FQRS complex wave in the indirect FECG signal. The second dataset is the B2 Labour dataset, denoted as the B2 dataset, comprising 12 records labeled “B2_Labour_X”, where X represents the record number. Each record includes 4 initially filtered AECG signals and 4 indirect FECG signals obtained after suppressing the MECG signals. Each signal spans 20 min, sampled at 500 Hz, and stored in LabView format in the binary file “B2_abSignals_X.ecg”. Furthermore, each record contains raw and preliminarily filtered FSE signals, each lasting 5 min with a sampling frequency of 1 kHz, stored in LabView format in the binary file “B2_dFECG_X.ecg”. Additionally, the file “B2_Maternal_R_X.txt” provides information about the maternal reference point, marking the position of the MQRS complex wave in the AECG signal. The file “B2_Fetal_R_X.txt” contains details regarding the fetal reference point, indicating the position of the FQRS complex wave in the FSE signal. The benchmark points in the B1B2 dataset underwent validation by clinical experts, resulting in each point being assigned an associated reliability flag. A flag of 0 signifies that the R-peak position could not be verified by the expert due to high signal interference, while a flag of 1 indicates successful verification of the R-peak position. Utilizing the annotations of the benchmark points within this dataset, both the fetal heart cycle (RR interval) and instantaneous heart rate (FHR) were precisely determined from the FECG signal.

The third database is the PhysioNet Fetal ECG Synthetic Database (FECGSYN), accessible at https://archive.physionet.org/physiobank/database/fecgsyndb/ (accessed on 30 August 2023) [30]. This database simulates adult and noninvasive fetal ECG signals using an electrocardiographic generative model. The model replaces maternal and fetal hearts with two point dipoles of varying spatial locations, shapes, and sizes on a spatial coordinate system. It synthesizes the abdominal ECG signal by treating each component in the abdominal ECG signal as independent. This approach allows for the provision of waveforms for each signal component. The database comprises 1750 synthesized signals in total, each sampled at a frequency of 250 Hz with a duration of 5 min.

#### 3.1.2. Database Description

The research presented in this paper relied on the utilization of the three aforementioned databases. However, owing to inconsistencies in the sampling frequencies across these databases, all the data were re-sampled to 500 Hz using fast Fourier transform. To streamline the training process of the neural network, the dataset was segmented with 1024 sample points serving as benchmarks. To ensure signal continuity, a 24-sample point overlap was introduced at the front and back of each pair of signals. The number of segmented samples for each database is outlined in Table 1.

To prevent data leakage, B2_Labour_dataset and 20% of ADFECGDB are allocated for the test set, while the remaining data are designated for the training set. The division is illustrated in Table 2.

### 3.2. Proposed Method

#### 3.2.1. Pre-Processing

The methods for acquiring NI-FECG signals involve notable sources of interference and noise [32]. Accurately extracting the FECG signal necessitates obtaining an AECG signal with a high signal-to-noise ratio. Consequently, the pre-processing of abdominal wall signals is imperative to mitigate baseline drift, power frequency interference, and pulse artifacts.

To eliminate the baseline drift, the signal is subjected to high-pass filtering. A low-pass first-order Butterworth filter with a cutoff frequency of 5 Hz is applied to estimate a baseline signal in the forward and backward directions. The baseline drift is then eliminated by subtracting the low-pass filtered signal from the original signal. Conventional methods typically employ high-order Butterworth bandpass filters to remove baseline drift and pulse artifacts. However, this approach often leads to phase lag issues and the potential emergence of challenging-to-eliminate ripple. In contrast, this paper presents an alternative approach by subtracting a low-pass signal to derive a high-pass filtered signal. This method effectively circumvents the phase lag problems associated with high-order Butterworth bandpass filters.

Addressing industrial power frequency interference involves the application of a trap filter. Initially, a forward–backward, zero-phase, and 1 Hz bandwidth trap filter is employed at the peak frequency and subsequent third harmonics. Subsequently, power frequency interference is assessed by comparing the peak power density near 50 Hz and 60 Hz with the average power density.

To mitigate the impulse artifacts, a moving median filter is applied to the signal. Initially, a moving median filter with a 60 ms window filters the signal to remove noise with impulse characteristics. Subsequently, the absolute difference between the original signal and the median-filtered signal is calculated, determining a threshold value. If the absolute difference exceeds this threshold, the signal is replaced with the average value of the interval signal.

After removing the aforementioned sources of noise, the AECG signals were centered and whitened to enhance the quality of the FECG signals. Initially, the AECG signals from each channel were centered by subtracting the average value of the AECG signals, resulting in zero-centered signals, per Equation (1), where x represents the mixing matrix of the abdominal wall source signals and $E\left\{x\right\}$ denotes the mean value of x:

(1) $$x_{c}=x-E\left\{x\right\}$$

Next, the signal undergoes a whitening process for decorrelation, as outlined in Equation (2), achieved through the eigenvalue decomposition of the covariance matrix. Here, V represents the orthogonal matrix of eigenvectors, and D denotes the diagonal matrix of eigenvalues. Through whitening, the original signal is decorrelated and orthogonalized, effectively reducing the number of parameters to be estimated.

(2) $$E\left\{x_{c}x_{c}^{T}\right\}=VDV^{T}$$

Finally, a whitening vector is created as depicted in Equation (3):

(3) $$x_{w}=VD^{-1/2}V^{T}x_{c}$$

**Figure 1:**
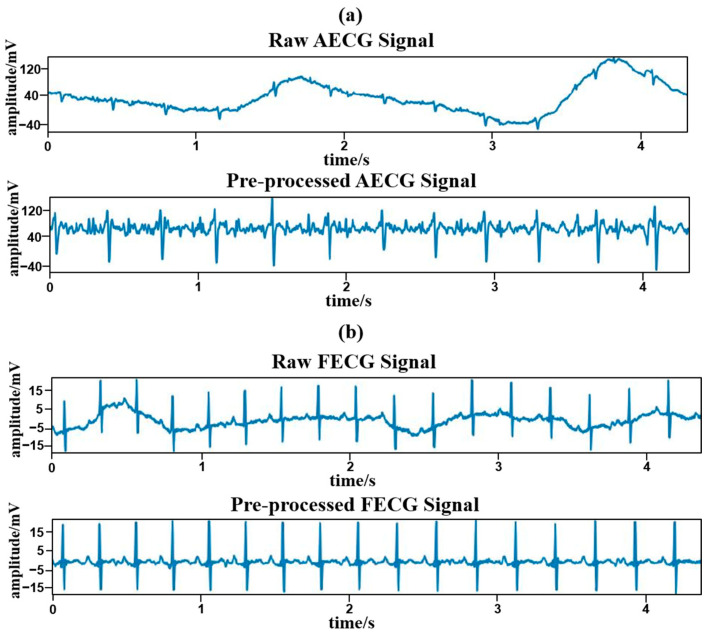
Signal before (**top**) and after (**bottom**) pre-processing. (**a**) Raw AECG signal and pre-processed AECG signal. (**b**) Raw FECG signal and pre-processed FECG signal.

A comparison between the raw signal and pre-processed signal is shown in Figure 1.

#### 3.2.2. Model Architecture

Here, we first describe the architecture of the main framework, followed by providing individual introductions to the architectures of the generator and discriminator.

##### Module Architecture

**Figure 2:**
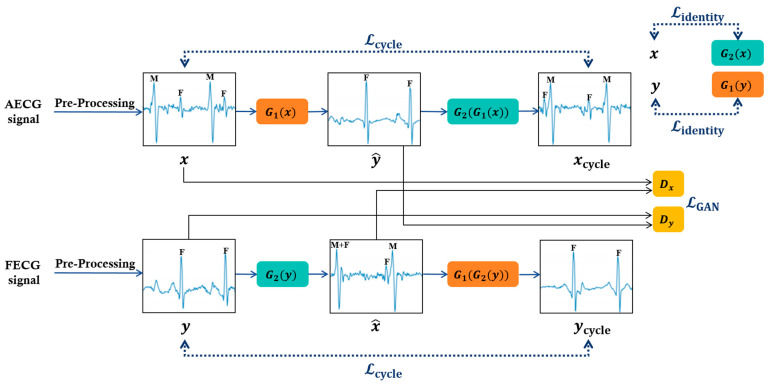
Training framework of the CycleGAN. Inputs are pre-processed signals. There are two generators ($G_{1},G_{2}$) and two discriminators ($D_{x}$ and $D_{y}$).

We employ an unsupervised learning approach using CycleGAN, as described in Figure 2. The input is the pre-processed signal. CycleGAN is essentially a paired network that learns two mappings using two generators: *G*_1_: AECG signal (*x*)→FECG signal (*y*) and G_2_:FECG signal (*y*)→AECG signal (*x*). Also, there are discriminators, *D_x_* and *D_y_*, for each signal domain, to compete with the generators. A generative adversarial network (GAN) is trained for each mapping. For one mapping, generator *G*_1_ is trained to generate an estimate of the FECG signal ($\hat{y}=G$_1_(*x*)), using the AECG signal (*x*) as input, which closely approximates the authentic FECG signal (*y*). Discriminator *D_y_* will classify the input as either a genuine FECG signal (*y*) or a synthetic FECG signal ($\hat{y}=G$_1_(*x*)) generated by the generator. For the other mapping, generator *G*_2_ is trained to generate an estimate of the AECG signal ($\hat{x}=G$_2_(*y*)), using the FECG signal (*y*) as input, which closely approximates the authentic AECG signal (*x*). Discriminator *D_x_* will classify the input as either a genuine AECG signal (*x*) or a synthetic AECG signal ($\hat{x}=G$_2_(*y*)) generated by the generator.

The training of CycleGAN is performed by solving the min–max problem for the generators and discriminators. The generators aim to minimize the loss function, while the discriminators aim to maximize it. This optimization problem can be expressed as follows:

- Add the cycle consistency loss ($L_{cycle}$) to maintain consistency between the two networks.

(4) $$\begin{array}{c} L_{cycle}(G_{1},G_{2})=E_{x\sim p_{data}(x)}{\left\|G_{2}(G_{1}(x))-x\right\|}_{1}\;+\\ E_{y\sim p_{data}(y))}{\left\|G_{1}(G_{2}(y))-y\right\|}_{1} \end{array}$$

Here, ${\left\|x\right\|}_{1}$ denotes the L1 norm of *x*, $G_{1}$ and $G_{2}$ should be inverse functions of each other, and $G_{1}$($G_{2}$(*x*)) = *x*; $G_{2}$($G_{1}$(*y*)) = *y*. This loss should be minimized in order to keep the two functions mutually inverse.

- Add the adversarial loss ($L_{GAN}$). Driven by the adversarial loss, the generator generates data with increasing fidelity and the discriminator with increasing discriminatory power.

(5) $$\begin{array}{c} L_{GAN}(G_{1},D_{y},X,Y)=E_{y\sim p_{data}(y)}[logD_{y}(y)]\;+\\ E_{x\sim p_{data}(x)}[log(1-D_{y}(G_{1}(x)))] \end{array}$$

(6) $$\begin{array}{c} L_{GAN}(G_{2},D_{x},X,Y)=E_{x\sim p_{data}(x)}[logD_{x}(x)]\;+\\ E_{y\sim p_{data}(y))}[log(1-D_{x}(G_{2}(y)))] \end{array}$$

Here, $E_{y\sim p_{data}(y)}[logD_{y}(y)]$ and $E_{x\sim p_{data}(x)}[logD_{x}(x)]$ represent the probability that the discriminator will determine the true data as true; $E_{x\sim p_{data}(x)}[log(1-D_{y}(G_{1}(x)))]$ and $E_{y\sim p_{data}(y))}[log(1-D_{x}(G_{2}(y)))]$ represent the probability that the discriminator will determine the data generated by the generator as false. Therefore, the total loss of the discriminator is the sum of the two, and that loss should be maximized.

- Add the identity loss to ensure that the generated FECG signals do not have unwanted distortions due to adversarial losses. The variation in the input signal is minimized by providing immobility constraints through the generator. Maximize this loss in order to ensure the morphological information of the FECG signal is available.

(7) $$\begin{array}{c} L_{identity}(G_{1})=\frac{2}{N}\sum_{i=1}^{N}\frac{1-\rho (P(y_{i}),P(G_{1}(x_{i})))}{\rho (P(y_{i}),P(x_{i}))}+\frac{4}{N}\sum_{i=1}^{N}1-\rho (y_{i},G_{1}(x_{i}))\;+\\ \frac{1}{N}\sum_{i=1}^{N}\left|\frac{p_{\left(x_{i}\right)}-p_{{(G}_{1}(x_{i}))}}{p_{\left(x_{i}\right)}}\right| \end{array}$$

(8) $$\begin{array}{c} L_{identity}(G_{2})=\frac{2}{N}\sum_{i=1}^{N}\frac{1-\rho (P(x_{i}),P(G_{2}(y_{i})))}{\rho (P(x_{i}),P(y_{i}))}+\frac{4}{N}\sum_{i=1}^{N}1-\rho (x_{i},G_{2}(y_{i}))\;+\\ \frac{1}{N}\sum_{i=1}^{N}\left|\frac{p_{\left(y_{i}\right)}-p_{{(G}_{2}(y_{i}))}}{p_{\left(y_{i}\right)}}\right| \end{array}$$

Here, P is the power spectral density, ρ is the Pearson correlation coefficient, $\rho (x,y)=\frac{\sum_{i=1}^{N}\left(x_{i}-\bar{x})(y_{i}-\bar{y}\right)}{\sqrt{\sum_{i=1}^{N}{\left(x_{i}-\bar{x}\right)}^{2}}\sqrt{\sum_{i=1}^{N}{\left(y_{i}-\bar{y}\right)}^{2}}}$, and p is the target signal power.

The total loss function is depicted in Equation (9):

(9) $$L(G_{1},G_{2},D_{x},D_{y})={\lambda L_{cycle}(G_{1},G_{2})+L}_{GAN}(G_{1},D_{y},X,Y)+L_{GAN}(G_{2},D_{x},X,Y)+L_{identity}(G_{1})+L_{identity}(G_{2})$$

λ regulates the relative significance of the three objectives, as illustrated in Equation (10):

(10) $${G_{1}}^{*},{G_{2}}^{*}=arg\underset{G_{1},G_{2}}{\min}\underset{D_{x},D_{y}}{\max}L(G_{1},G_{2},D_{x},D_{y})$$

##### Generator

**Figure 3:**
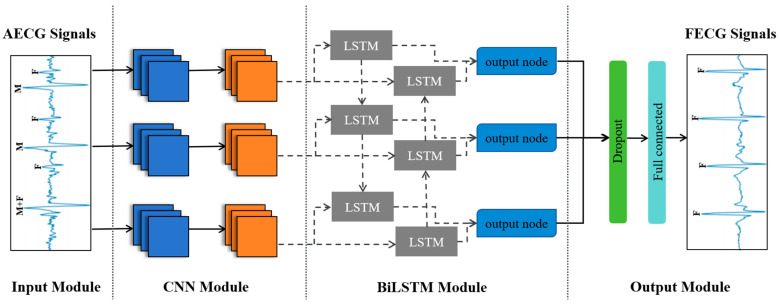
Generator based on combined CNN–BiLSTM structure.

The CNN–BiLSTM generator consists of four parts, the signal input module, convolutional neural network module, BiLSTM module, and output module, as described in Figure 3. The last three parts correspond to the three functions of morphological feature extraction, feature enhancement, and fetal ECG signal reconstruction, respectively. The input module takes the AECG signal as input, extracts combined features through CNN and BiLSTM, and transforms the feature vectors in the output module to reconstruct the FECG signal that is of equal length to the input signal. The generator extracts high-dimensional features with the assistance of the CNN’s ability to abstract short-sequence features. Subsequently, BiLSTM synthesizes the short-sequence high-dimensional features to perform time series prediction, making it well suited for processing time series data with local correlations. The network structure can efficiently capture deep combinatorial features containing morphological features and temporal dependencies. Using this depth feature, the CNN–BiLSTM generator can better separate the FECG signal from the rest and complete FECG signal reconstruction with less information loss.

**Figure 4:**
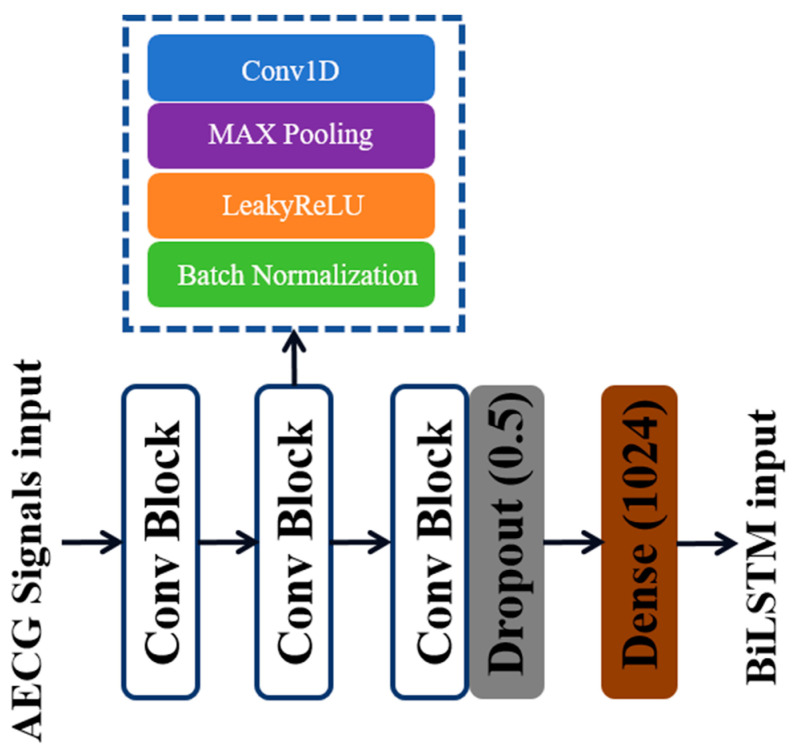
Schematic diagram of single-layer CNN network structure.

**Morphological feature extraction:** Although there are multiple similarities between the FECG signal and other parts in the AECG signal, the diversity of morphological features in the time domain can serve as a typical feature with which to distinguish them. In this paper, a one-dimensional convolutional neural network is used to extract the morphological features of FECG signals from AECG signals. When the signal passes through the convolutional layer, one-dimensional convolution is employed to extract the corresponding morphological features. The network is then expanded both horizontally and vertically to acquire deeper and more abundant features. That is, the number of convolution blocks is increased to 3, making 3 layers of convolution layers parallel. The specific structures of each convolutional block and pooling block are described in Figure 4. Finally, the dropout layer and fully Connected layer are added to prevent overfitting and enhance feature depth, and the size is adjusted to serve as input for the BiLSTM module. The number of convolution kernels in each convolutional block increases by a multiple of 2, ranging from 64 to 256, progressively extracting features and enhancing feature dimensions. The second part of the convolutional neural module is the pooling layer, which performs feature dimensionality reduction, eliminates redundant features, and improves the fault tolerance of the CNN structure. After completing spatial feature extraction, the feature vectors obtained from three CNN modules are input into the BiLSTM network.

**Figure 5:**
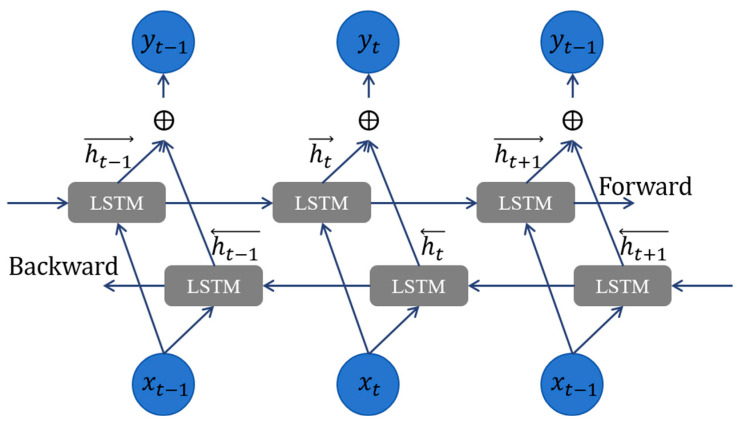
Specific structure of BiLSTM networks.

**Figure 6:**
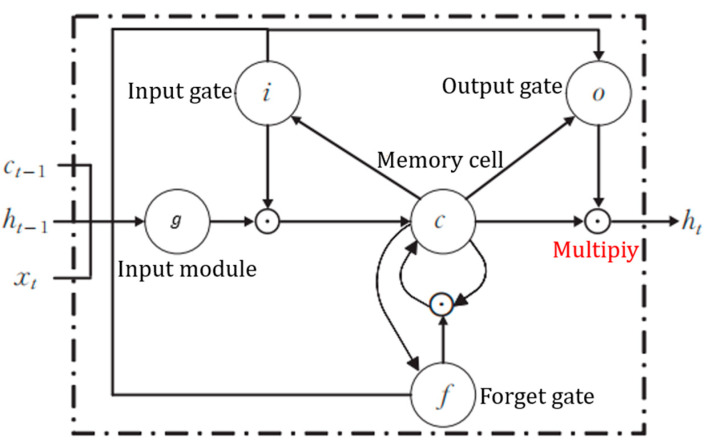
Internal structure of the LSTM module.

**Feature enhancement:** This study utilizes bidirectional long short-term memory (BiLSTM) networks to learn signal timing information, reinforcing the feature differences between FECG signals and other components of the AECG signal. The specific structures of the BiLSTM and LSTM modules are described in Figure 5 and Figure 6. The main hidden layer structure of the BiLSTM network is composed of an LSTM network with forward input operation and an LSTM network with reverse input operation based on the LSTM network. While retaining the characteristics of the LSTM unit structure, it pays more attention to the correlation between temporal data and ensures the extraction of time series features by continuously adjusting the number of BiLSTM hidden layers to explore the optimal time series feature extraction mode. The hidden layer includes three output nodes, corresponding to the feature outputs of the P-wave, QRS compound wave, and ST segment of the FECG signal. The feature vectors are transformed into combined features containing signal properties and temporal dependencies after passing through the BiLSTM module.

**Fetal ECG signal reconstruction:** The combined feature vectors are transformed into segments of FECG signals with an equal length to that of the AECG signal through the fully connected layer at the end.

##### Discriminator

**Figure 7:**
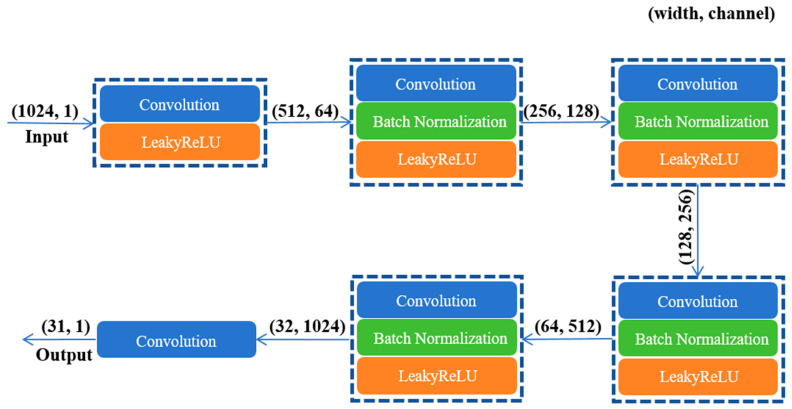
Discriminator based on PatchGAN structure.

Applying a 4 × 4 PatchGAN as the discriminator for the CycleGAN, in contrast to a regular GAN discriminator, which maps the input to a single real number representing the probability that the input signal is a genuine FECG signal, the PatchGAN discriminator maps the input to a 4 × 4 matrix. The value $X_{ij}$ in the matrix represents the probability that each patch is a genuine sample. The discriminator’s final output is obtained by averaging the values of $X_{ij}$. The PatchGAN discriminator discriminates each small segment of the signal, directing the training model to focus more on the details of the signal. The discriminator structure is shown in Figure 7. In the discriminator, convolution layers with a kernel size of 2 were used. Instead of ReLU being used as the activation function, LeakyReLU with an α value of 0.2 was used. During training, the discriminator receives a patch and reduces the size of the feature map by half using the convolution layer with strides of 2. The number of channels starts from 64 and increases 2-fold. The last convolution layers use strides of 1.

### 3.3. Learning Process for FECG Signal Extraction Model

The process of extracting the FECG signal from the AECG signal can be regarded as a reconstruction of the FECG signal. Let $y\in R^{N\times 1}$ be the FECG signal following the distribution *F*(*y*), and $x\in R^{N\times 1}$ be the FECG signal to be reconstructed (AECG signal) following another distribution *F*(*x*). Here, N represents the length of data segments. The objective of the model is to build a function, $G_{1}(\theta )$, that maps *x* to *y*:

(11) $$G_{1}(\theta ):x\to y$$

The reconstruction model is completed when the parameter set *θ* minimizes the difference between *F*($G_{1}(x)$) and *F*(*y*).

There is no explicit distribution mapping relationship between the AECG signal and the FECG signal. GAN learns deep features that can describe the distributions of both AECG and FECG signals. Using this information, it accomplishes the aforementioned distribution mapping, achieving the reconstruction of FECG signals with lower non-linear information loss. GAN takes AECG signals as input, and each layer of the network learns features from the feature vectors generated by the input layer or the previous layer, generating deeper features for subsequent network layers.

The pre-processed AECG signal and the real FECG signal are used to train and optimize the model. The real FECG signal is used as a learning objective to approximate the total model loss function, which is *F*($G_{1}(x)$) to *F*(*y*).$L_{total}(G_{1},G_{2},D_{x},D_{y})=-L_{GAN}+\alpha L_{cycle}+\beta L_{identity}$, where α and β denote the loss weights for the cycle loss and the identity loss, respectively.

In addition, model optimization was performed using the Adam optimizer. Hyper-parameters for training the network are described in Table 3.

### 3.4. Evaluation Methods

In order to validate the model’s performance in extracting FECG signals, the mean square error (MSE), the mean absolute error (MAE), the R-squared goodness of fit ($R^{2}$), and the signal-to-noise ratio (SNR) are exploited to evaluate the quality of the extracted FECG signal, defined as follows:

(12) $$MSE=\frac{1}{N}\sum_{n=1}^{N}{\left[F(G_{1}(n))-F(n)\right]}^{2}$$

(13) $$\;MAE=\frac{1}{N}\sum_{n=1}^{N}\left[F(G_{1}(n))-F(n)\right]$$

(14) $$R^{2}=1-\frac{\sum_{n=1}^{N}{\left[F(G_{1}(n))-F(n)\right]}^{2}}{\sum_{n=1}^{N}{\left[\bar{F(n)}-F(n)\right]}^{2}}$$

(15) $$\;SNR=10\log_{10}\frac{\sum_{n=1}^{N}{\left[F(G_{1}(n))\right]}^{2}}{\sum_{n=1}^{N}{\left[F(G_{1}(n))-F(n)\right]}^{2}}$$

where  F(n) is the real FECG signal, $F(G_{1}(n))$ is the FECG signal generated by the generator, and $\bar{F(n)}$ is the average value of the real FECG signal. Smaller values of MSE and MAE signify a better model fit, larger $R^{2}$ values indicate higher correlation, and increased SNR values reflect a higher quality of the extracted FECG signal.

The reference QRS compound wave annotation is usually used in fetal ECG signal extraction to illustrate the model’s performance by comparing the position of the QRS compound wave with the detected QRS compound wave position in the extracted FECG signals, and the improved Pan–Tompkins detection algorithm [33] is used for FQRS compound wave detection from the extracted FECG signals. If the position of the QRS compound wave in the extracted FECG signal differs by no more than 50 ms from the reference position, it is considered to be extracted correctly. To validate the model’s performance in extracting FQRS compound waves, the sensitivity (Se), the positive predictive value (PPV), the accuracy (ACC), and F1 are exploited to evaluate the quality of the extracted FQRS compound wave, defined as follows:

(16) $$Se=\frac{TP}{TP+FN}\times 100\%$$

(17) $$\;PPV=\frac{TP}{TP+FP}\times 100\%$$

(18) $$ACC=\frac{TP}{TP+FN+FP}\times 100\%$$

(19) $$F_{1}=2\times \frac{Se\times PPV}{Se+PPV}=\frac{2TP}{2TP+FN+FP}\times 100\%$$

where TP, FP, and FN represent the quantities of true positives (a correctly detected FQRS compound wave), false positives (an incorrectly detected FQRS compound wave), and false negatives (missed detections of an FQRS compound wave), respectively. Higher values of Se, PPV, ACC, and $F_{1}$ metrics indicate better performance of the FECG signal extraction algorithm.

## 4. Results

In this section, the model’s performance is comprehensively illustrated through the evaluation of the quality of extracted FECG signals and the detection of FQRS compound wave extraction accuracy. Finally, a model ablation study is conducted to demonstrate the optimality of the model structure.

### 4.1. FECG Signal Extraction Quality Assessment

Firstly, the FECG signal extraction performance of the CycleGAN combined CNN–BiLSTM architecture (CBLS-CycleGAN) is assessed on B2_Labour_dataset. As outlined in Table 4, CBLS-CycleGAN achieves an MSE of 0.027, MAE of 0.012, $R^{2}$ of 98.53%, and SNR of 7.45.

Next, a comparison is made between the approach presented in this paper and six other FECG signal extraction algorithms using the ADFECGDB dataset. As summarized in Table 5, the CBLS-CycleGAN demonstrates superior performance with an MSE of 0.019, MAE of 0.006, and $R^{2}$ of 98.01%. Notably, models leveraging the CycleGAN as a foundational framework outperform other models, underscoring the high-quality extraction of FECG signals by the GAN.

**Figure 8:**
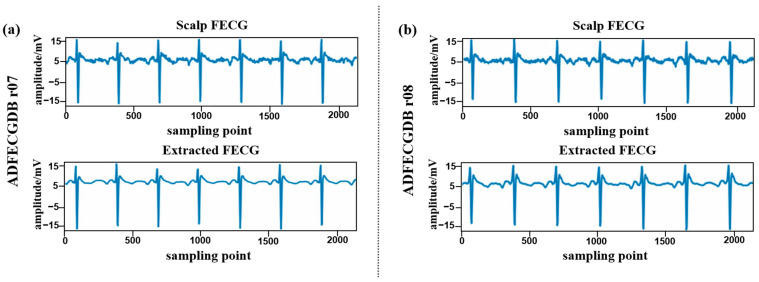
Visualized example of the proposed model’s FECG signal extraction performance when using ADFECGDB. Above is the scalp FECG signal, and below is the extracted FECG signal. (**a**) ADFECGDB r07; (**b**) ADFECGDB r08.

**Figure 9:**
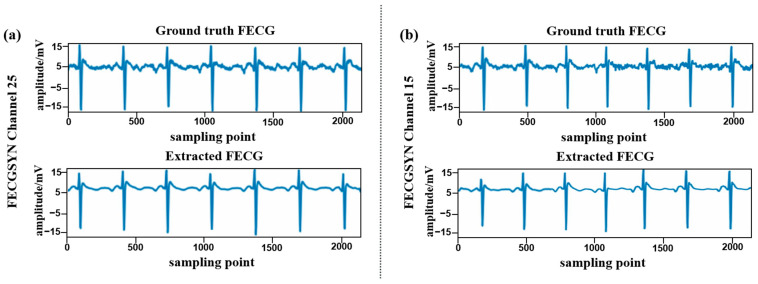
Visualized example of the proposed model’s FECG signal extraction performance when using FECGSYN. Above is the ground truth FECG signal, and below is the extracted FECG signal. (**a**) FECGSYN25; (**b**) FECGSYN15.

Figure 8 illustrates the prediction of the two signals from ADFECGDB, and Figure 9 illustrates the prediction of the two signals from FECGSYN. Visual examination reveals that the extracted FECG signals closely resemble the scalp FECG signals, exhibiting superior recovery of detailed features associated with small amplitudes at low frequencies and preserving the morphological information of FECG signals.

**Figure 10:**
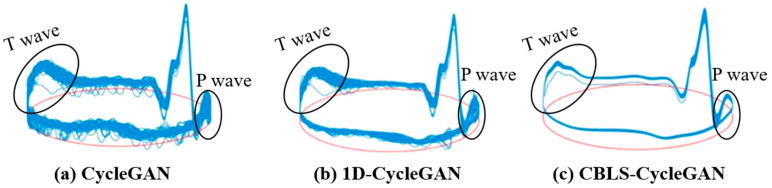
Phase envelopes of FECG signals obtained through extraction using various CycleGAN models.

Finally, Figure 10 illustrates an image comprising a unit circle (depicted in red) alongside a 3D trajectory (depicted in blue) generated based on data from ADFECGDB r01. As the trajectory approaches one of the P-QRS-T waves, the 3D trajectory exhibits vertical movement, with the limit ring oscillating up and down. The projection of this 3D trajectory onto the *Z* axis corresponds to the FECG signal. A visualization of the 3D trajectories clearly demonstrates that the FECG signal cycles extracted using the CBLS-CycleGAN model exhibit the strongest cycle consistency and allow the complete preservation of P-QRS-T morphological information, surpassing both the traditional CycleGAN model and the 1D-CycleGAN model.

### 4.2. FQRS Compound Wave Detection Evaluation

Initially, using the improved Pan–Tompkins algorithm for the FQRS compound wave detection of FECG signals extracted from the CBLS CycleGAN, the performance of FQRS compound wave detection by the CBLS-CycleGAN is evaluated on the ADFECGDB database. As detailed in Table 6, the CBLS-CycleGAN achieves an Se of 99.34%, PPV of 99.31%, and *F*_1_ of 99.33%. Despite significant noise pollution in this database, the CBLS-CycleGAN model demonstrates robust FQRS compound wave extraction. The model successfully captures a substantial number of FQRS compound waves with fewer instances of both missed and falsely detected FQRS compound waves, providing further evidence of the model’s validity in challenging conditions.

Next, based on a variety of databases, the FQRS compound wave detection results of the CBLS-CycleGAN are compared with the FQRS compound wave detection results of the other eight deep learning models, as shown in Table 7. The CBLS-CycleGAN exhibits the best FQRS compound wave detection performance on B2_Labour dataset; it achieves an Se of 99.67%, PPV of 99.82%, and *F*_1_ of 99.74%. Compared with the traditional single CycleGAN model, the model’s performance is highly improved because the present model preserves both the spatial and temporal features of the signal. On the ADFECGDB dataset, the performance parameters of the present model are slightly lower than those of CAA-CycleGAN, but the model is based on the attention mechanism, which has higher computational complexity and is time-consuming, which is not conducive to the real-time monitoring of fetal health status.

**Figure 11:**
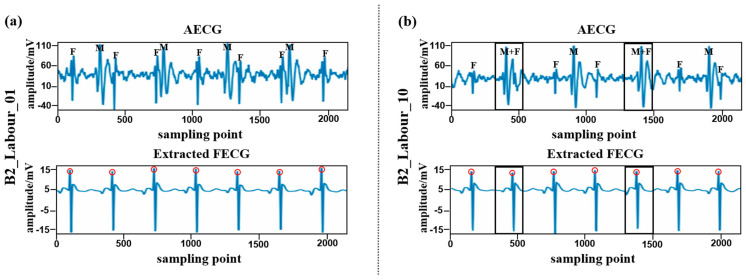
Visualized example of the proposed model’s FQRS compound wave extraction performance when using B2_Labour_dataset. Above is the AECG signal, and below is the extracted FECG signal. The positions of the FECG signal, MECG signal, and MECG signal overlapping with the FECG signal in the AECG signal are indicated by ‘F’, ‘M’, and ‘F + M’. The R peaks detected by the improved Pan–Tompkins algorithms are marked with red circles. (**a**) B2_Labour_01; (**b**) B2_Labour_10.

Finally, Figure 11 illustrates an example of the proposed model’s visualization of FQRS compound wave extraction performance when utilizing B2_Labour_dataset. Visual inspection indicates that this model adeptly segregates FECG signals from MECG signals, thereby retaining a greater degree of morphological information. Even in scenarios where there is overlap between maternal fetal electrocardiogram signals (as denoted by the black box), this model reliably yields clear FECG signals.

### 4.3. Ablation Study

**Figure 12:**
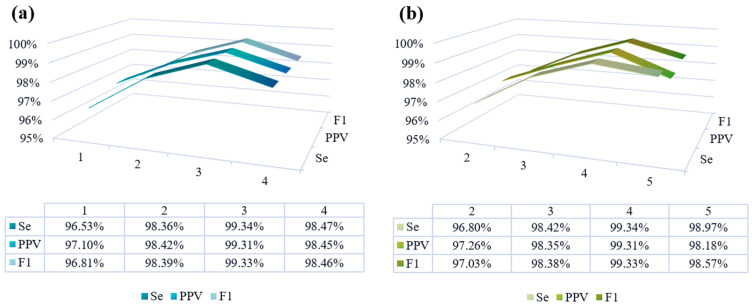
Ablation experiments were conducted on the generator and discriminator network depths using the ADFECGDB database. An experimental comparison of CNN generators with varying numbers of layers is presented on the (**a**), while the (**b**) side showcases an experimental evaluation of PatchGAN discriminators with different layer depths.

The generator in this framework employs a combination of CNN and BiLSTM layers. An increased number of CNN layers signifies greater model depth, facilitating superior nonlinear representation and enabling the learning of more complex mappings. On the other hand, the discriminator utilizes a PatchGAN architecture, where additional PatchGAN layers enhance the discriminator’s focus on signal details. However, excessively deep networks can escalate computational demands and potentially trigger overfitting issues, thereby compromising FECG signal extraction accuracy. To pinpoint the optimal number of generator and discriminator layers, FECG signal extraction experiments were conducted on ADFECGDB data using models with varying layer configurations. As illustrated in Figure 12, the Se, PPV, and *F*_1_ value indicate that the network attains optimal performance when employing three CNN layers and four PatchGAN layers.

To assess the impact of different modules within the CBLS-CycleGAN on network performance, this study incorporates two types of generators and two types of discriminators for combination. The results are summarized in Table 8. Notably, optimal network performance is observed when employing the CNN–BiLSTM generator in conjunction with the PatchGAN discriminator.

## 5. Conclusions

To address the issue of existing algorithms failing to preserve the morphological features of fetal ECG signals, we have developed a novel CycleGAN architecture, the generator of which combines the spatial features extracted by the convolutional neural network and the temporal features extracted by the BiLSTM network, and designs three hidden output nodes corresponding to the waveform features of the FECG signals. The discriminator, known as PatchGAN, discriminates each small segment of the signal, enhancing the model’s focus on signal details during training. Ultimately, our implementation, employing CBLS-CycleGAN, achieves the reconstitution of FECG and MECG from AECG data with minimal information loss. The CBLS-CycleGAN model showcases exceptional preservation of signal morphology while achieving performance on par with that of the state-of-the-art methods. Moreover, it significantly enhances the accuracy of FQRS complex wave extraction.

The validation of the proposed method in this study, using two publicly available real databases, demonstrates that the model accurately acquires the FQRS compound wave of the signal. With the Se, PPV and *F*_1_ of 99.51%, 99.57%, and 99.54%, respectively, based on ADFECGDB and B2_Labour, the model showcases high performance. Moreover, it efficiently preserves the morphological information of the FECG signal, as indicated by the MSE, MAE, and *R*^2^ of 0.019, 0.006, and 98.01, respectively, based on ADFECGDB. Subsequent ablation experiments were conducted to validate the robustness and reliability of the model by varying the model’s depth and removing key components.

The work presented in this paper offers valuable clinical insights for the early diagnosis and intervention of fetal anomalies. Moving forward, it is essential to gather an extensive amount of real clinical data under the supervision of physicians to evaluate the CBLS-CycleGAN effectively, particularly in the context of remote home monitoring. In the future, enhancements to the CBLS-CycleGAN model could involve incorporating an attention layer before the random inactivation layer. The attention module aims to compute the weighted average sum of the output vectors from the last layer of the LSTM. By integrating this module, the model can amplify the impact of memory nodes with the highest weights in the Bi-LSTM, thereby further minimizing model errors.

## Figures and Tables

**Table 1:** The number of samples in each database after signal segmentation.

Database	The Number of Samples
ADFECGDB	1480
B1_Pregnancy_dataset	5920
B2_Labour_dataset	3552
FECGSYN	20,000

**Table 2:** The division of the training and test sets.

Dataset	The Number of Samples
training set	27,104
test set	3848

**Table 3:** Hyperparameters for the training network in the proposed framework.

Hyperparameter	Value
Optimizer	Adam
Initial learning rate	$10^{-4}$
$\beta_{1}$	0.9
$\beta_{2}$	0.999
Training rounds	80

**Table 4:** Evaluating the quality of the extracted FECG signals based on B2_Labour_dataset.

Data	MSE	MAE	$R^{2}$	SNR
B2_Labour_01	0.024	0.018	97.64	7.48
B2_Labour_02	0.028	0.010	98.57	7.58
B2_Labour_03	0.027	0.012	98.62	7.61
B2_Labour_04	0.025	0.009	99.01	7.47
B2_Labour_05	0.030	0.009	97.96	7.32
B2_Labour_06	0.028	0.010	97.47	7.02
B2_Labour_07	0.027	0.012	99.57	7.77
B2_Labour_08	0.027	0.013	98.71	7.31
B2_Labour_09	0.029	0.011	98.65	7.44
B2_Labour_10	0.031	0.017	99.41	7.47
B2_Labour_11	0.024	0.007	98.01	7.49
B2_Labour_12	0.024	0.012	98.74	7.44
MEAN ± STD	0.027 ± 0.002	0.012 ± 0.003	98.53 ± 0.63	7.45 ± 0.18

**Table 5:** Comparison of FECG signal extraction quality with that of existing techniques based on ADFECGDB. (MEAN ± STD).

Method	MSE	MAE	$R^{2}$
${PA}^{2}NET$ [34]	0.146 ± 0.014	0.098 ± 0.007	79.87 ± 0.35
RCED-Net [24]	0.061 ± 0.006	0.019 ± 0.005	90.69 ± 0.17
AEDL [35]	0.059 ± 0.002	0.018 ± 0.003	92.09 ± 0.22
CSGSA-Net [36]	0.057 ± 0.003	0.016 ± 0.002	92.27 ± 0.33
CycleGAN [27]	0.042 ± 0.008	0.011 ± 0.004	92.71 ± 0.29
CAA-CycleGAN [28]	0.024 ± 0.003	0.007 ± 0.002	95.34 ± 0.12
this work	0.019 ± 0.004	0.006 ± 0.002	98.01 ± 0.26

**Table 6:** Evaluating model FQRS compound wave detection performance with five datasets from the ADFECGDB database.

Data	TP	FN	FP	Se (%)	PPV (%)	ACC (%)	*F*_1_ (%)
r01	637	7	5	98.91	99.22	98.15	99.07
r07	626	1	2	99.84	99.68	99.52	99.76
r10	651	6	7	99.09	98.94	98.04	99.01
r04	628	4	3	99.37	99.52	98.90	99.45
r08	639	3	5	99.53	99.22	98.76	99.38
SUM	3181	21	22	99.34	99.31	98.67	99.33

**Table 7:** Comparison of FQRS compound wave detection quality with that of existing techniques.

Method	Database	Se (%)	PPV (%)	*F*_1_ (%)
RCED-Net [24]	ADFECGDB	96.06	92.25	94.10
	PCDB	92.60	94.68	93.62
DPSS [37]	FECGSYNDB	98.55	99.52	99.03
	ADFECGDB&NIFECGDB	95.75	97.29	96.50
${PA}^{2}NET$ [34]	ADFECGDB	99.48	99.74	99.61
	NIFECGDB	99.58	99.67	99.62
AEDL [35]	NIFECGDB	97.36	98.68	98.02
CSGSA-Net [36]	ADFECGDB	99.61	99.44	99.56
	B2_Labour	99.61	98.91	98.79
CycleGAN [27]	ADFECGDB	99.46	99.67	99.56
	NIFECGDB	96.89	97.26	97.07
1D-CycleGAN [29]	ADFECGDB&B2_Labour	97.69	94.87	96.26
CAA-CycleGAN [28]	FECGSYNDB	98.37	97.78	98.16
	ADFECGDB	99.67	99.64	99.71
	B2_Labour	99.54	99.43	99.47
**this work**	FECGSYNDB	99.70	98.46	98.76
	ADFECGDB	99.34	99.31	99.33
	B2_Labour	**99.67**	**99.82**	**99.74**

**Table 8:** Ablation studies for the proposed modules in CBLS-CycleGAN tested on the ADFECGDB database.

Generator		Discriminator							
CNN	Bi-LSTM	Basic	PatchGAN	MSE	MAE	$R^{2}$	Se	PPV	$F_{1}$
√		√		0.043	0.019	92.47	95.11	96.02	95.56
	√	√		0.047	0.020	91.44	94.21	95.48	94.84
√	√	√		0.034	0.011	94.56	96.07	97.77	96.91
√			√	0.021	0.009	97.32	98.57	98.49	98.53
	√		√	0.024	0.010	96.10	97.99	98.01	98.00
√	√		√	0.019	0.006	98.01	99.34	99.31	99.33

## Data Availability

The data used to support the findings of this study are included in the article.
